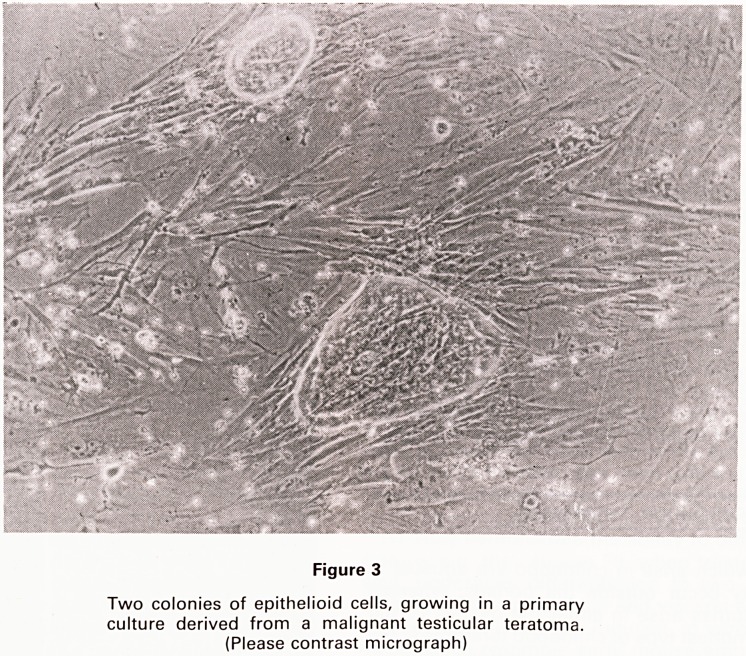# Cellular Biology of Teratoma

**Published:** 1988

**Authors:** K. W. Brown, V. Poirier, S. M. Tyler, N. J. Maitland

**Affiliations:** Department of Pathology, The Medical School, Bristol University; Department of Pathology, The Medical School, Bristol University; Department of Pathology, The Medical School, Bristol University; Department of Pathology, The Medical School, Bristol University


### Cellular biology of teratoma

**K. W. Brown, V. Poirier, S. M. Tyler, and N. J. Maitland**
Department of Pathology, The Medical School, Bristol University

### INTRODUCTION

Teratomas are bizarre tumours which contain multiple tissues of kinds foreign to the part of the body in which they arise. Within teratomas these tissues can be organised into recognisable organs, limbs and, in some instances, structures which resemble a complete fetus (1,2,3). These 'monstrous' features give these tumours their name (teras=monster, in Greek). A common example of this bizarre differentiation is the production of hair and skin within benign ovarian teratomas (Fig. 1).

The nature of teratomas is due to the fact that they arise from pluripotent cells, i.e. early enbryonic cells or primordial germ cells. Thus a teratoma can contain tissues which are derived from all three embryonic cell layers, and this constitutes the strictest definition of a teratoma (1). If the pluripotent stem cells within a teratoma all undergo differentation to fully mature tissues, then the result is a benign tumour. Persistence of the stem cells within the teratoma results in a malignant tumour. Malignancy may also occur as a consequence of malignant transformation occurring in the differentiated tissues within a teratoma.

**Figure 1**

Histological section of a benign ovarian teratoma (H & E stain). Within this field are visible hairs (H) and areas of epidermis (E)

### MOUSE TERATOMAS AND TERATOMA CELL LINES

Certain strains of mice are susceptible to developing teratomas, and teratomas can also be induced experimentally, by implanting tissue from fetal gonads or early embryos, into suitable sites on a host animal (4,5). The malignant teratomas of mice are normally referred to as teratocarcinomas, and the pluripotent stem cell of these tumours has been named the embryonal carcinoma (EC) cell. EC cells have been cultured from teratocarcinomas, and there are now many EC cell lines available, which possess several intriguing properties:

(1) They demonstrate the ability to differentiate in cell culture, e.g. the cell line F9, when treated with retinoic acid, undergoes differentiation to two types of extra-embryonic tissue; parietal and visceral endoderm (6);

(2) some lines can be induced, *in vitro*, to form structures which resemble early mouse embryos, known as embryoid bodies (7);

(3) when injected into syngeneic hosts, the EC cells produce malignant teratocarcinomas (6,7) and;

(4) if EC cells are injected into early mouse embryos and these are subsequently implanted into pseudopregnant host mothers, then chimeric mice are eventually born. In these chimeras, the EC cells have actually differentiated to form normal (non-malignant) tissues, i.e. they can participate in normal embryonic development (7).

The ability of EC cells to form embryoid bodies, and to participate in normal embryogenesis during the production of chimeric mice, suggests that EC cells are closely related to early embryonic cells. It has, in fact, been suggested that EC cells are normal embryonic cells, which display malignant potential when placed in an inappropriate environment (5,7). This close relationship between EC cells and early embryonic cells has recently been further strengthened, by the finding that cell lines can be developed from normal mouse embryos. These cell lines, known as EK cells, behave essentially identically to EC cells (8).

It is therefore apparent that, given the right conditions, the stem cells from mouse teratocarcinomas can either form malignant tumours, or alternatively differentiate into normal cells. Many studies have now been carried out, which aim to discover the molecular events which control the differentiation of mouse EC cells. In particular, it has been demonstrated that thedifferentiation of EC cells is accompanied by changes in the expression of proto-oncogenes (e.g. c-fos (9)) and also of homeo box-containing genes (10,11). It is still a matter of controversy as to whether these changes are causal, or merely represent alterations which are secondary to other undiscovered molecular events.

### HUMAN TERATOMA CELL LINES

Most of the human cell lines produced so far have been derived from malignant adult testicular teratomas. These cell lines retain a malignant phenotype, as demonstrated by their ability to produce malignant teratomas, when injected into nude (athymic) mice. However, the majority of such tumours are not highly differentiated, and in most cases the cell lines have proved extremely difficult to induce to differentiate *in vitro* (12,13). The exceptions to this appear to be the clones recently derived from the testicular teratoma cell line Tera-2 (14,15). These clones produce malignant teratomas in nude mice, and extensive somatic differentiation is observed in the tumours (14,15). In addition, the clones can be induced to differentiate into many cell types *in vitro*, and in particular neuronal differentiation has been observed (15, 16). However, these clones have not been shown to differentiate into extra-embryonic tissues, i.e. they cannot produce tissues which are characteristic of the earliest stages of embryonic development, and so it is doubtful whether these clones are truly pluripotent stem cells. Nonetheless, the Tera-2 derivatives are the nearest human equivalent to mouse EC cells at present available.

### CHILDHOOD TERATOMAS

The teratomas which arise in childhood are unusual in that the majority occur outside the gonads, whereas most adult teratomas arise in either the ovaries or the testes. The commonest type of childhood teratoma is the sacrococcygeal teratoma, which constitutes approximately 45% of all cases, the remainder being ovarian (30%), testicular (6%) and other sites (19%) (2). The sacrococcygeal teratomas have an incidence of approximately 1 in 40,000 births, and many cases are recognised at birth since the tumours often present as large extrapelvic growths. When discovered at birth, sacrococcygeal teratomas are almost always benign, but the incidence of malignancy increases with age of diagnosis; which may indicate that most of these tumours possess a strong potential for malignant transformation (2). Ashcraft and his colleagues have reported several cases in which sacrococcygeal teratomas show an apparent autosomal dominant pattern of inheritance (17). It is therefore possible that there are strong genetic factors which predispose individuals to develop these tumours, as has been found in other childhood tumours such as retinoblastoma and Wilms' tumour.

### EXPERIMENTAL STUDIES ON CHILDHOOD TERATOMAS

Our own work has been directed towards attempting to culture cell lines from childhood teratomas, since this class of childhood tumour has remained relatively neglected in terms of studying its cellular and molecular biology.

We have cultured samples from teratomas using standard tissue culture methods; briefly, pieces of tissue are minced and then fragments are placed in plastic petri dishes in a standard culture medium, supplemented with fetal calf serum. In addition, a feeder layer of non-dividing mouse fibroblasts is used, since this has been previously shown to promote the growth of many mouse teratoma cell lines. After a period of days to weeks, cells grow out from the tissue explants, and when the areas of cells are large enough, they can be removed with tryspin and transferred to a new culture dish. In this way, the many different cell types arising from a single tumour can be cultured separately.

**Figure 2**

Phase contrast micrographs of four morphologically different cell types, cultured from a benign sacrococcygeal teratoma

An example of the results of this approach is shown in Fig. 2, which shows cells of four distinctly different morphologies that have been derived from a benign sacrococcygeal teratoma. Immunohistochemical staining with monoclonal antibodies to intermediate filaments is routinely used to characterize such cells, since intermediate filaments are specific markers for different cell types (18). From the benign sacrococcygeal teratoma, we have cultured populations of cells which produce vimentin and keratin, and in primary cultures a small number of cells have been observed which produce either desmin or neurofilament proteins (C. Holmes, personal communication). Thus many diverse cell types have been cultured from a benign sacrococcygeal teratoma, consistent with the extensive somatic differentiation which was observed on standard histological examination. Likewise, we have observed many different cell types in cultures derived from two benign ovarian teratomas.

In benign teratomas, all the stem cells would have differentiated to fully mature tissues, and therefore, it would not be expected that immortal stem cell lines could be derived from such tumours. However, malignant teratomas would be expected to contain such cells. So far, we have been unable to produce any immortal cell lines from the two specimens that we have received from malignant teratomas. Epithelial cells, with the expected morphology for teratoma stem cells, have been observed in cultures derived from a malignant testicular teratoma (Fig. 3), but these cells did not form an established cell line. Few conclusions can be made from the small number of malignant teratomas that we have cultured, except that the production of stem cell lines is obviously not a simple procedure. The failure so far

**Figure 3**

Two colonies of epithelioid cells, growing in a primary culture derived from a malignant testicular teratoma. (Please contrast micrograph)

could be due either to inappropriate conditions of tissue dispersion or culture, or due to there being very few viable stem cells within the portions of tumour which have been cultured.

In future, we hope to increase the number of samples cultured, by obtaining samples from several other U.K. paediatric oncology centres. In addition, we aim to grow childhood teratomas as xenografts in nude mice, which would then provide a long term source of teratoma tissue, which could be used to optimize tissue culture conditions. The development of permanent cell lines from childhood teratomas will provide the necessary experimental material with which to investigate the cellular and molecular biology of these tumours.

### ACKNOWLEDGEMENTS

The authors thank Mr A. Skuse for the histology, Mr C. Jeal and Mrs S. Hagin for the photography, and Mrs J. Gilbert and Ms J. McRill for typing the manuscript.

This work was supported by the Cancer and Leukaemia in Childhood Trust.

### REFERENCES

1. ASHLEY, D. J. B. (1978) *in* Evan's histological appearance of tumours, 3rd edn, Churchill Livingstone, Edinburgh, pp 845–857.
2. GONZALEZ-CRUSSI, F. (1982) Extragonadal teratomas, Atlas of tumour pathology, 2nd series, Fascicle 18, Armed forces institute of pathology, Washington.
3. SCHWEISGUTH, O. (1982) Solid tumors in children, John Wiley & Sons, New York, pp 157–166.
4. STEVENS, L. C. (1983) *in* Teratocarcinoma stem cells, eds. Silver, L., Martin, G. R. and Strickland, S., Cold Spring Harbor, New York, pp 23–36.
5. SOLTER, D. (1983) *in* The human teratomas. Experimental and Clinical Biology, eds. Damjanov, I., Knowles, B. B. and Solter, D., Humana Press, New Jersey, pp 343–356.
6. STRICKLAND, S. (1981) *Cell*, **24**, 277–278.
7. MARTIN, G. R. (1980) *Science*, **209**, 768–776.
8. EVANS, M. *et al.* (1983) *in* Current problems in germ cell differentiation, eds. McLaren, A. & Wylie, C. C., Cambridge University Press, Cambridge, pp 139–155.
9. MASON, I. *et al* (1985) Differentiation, **30**, 76–81.
10. MANLEY, J. L. and LEVINE, M. S. (1985) *Cell*, **43**, 1–2.
11. SNOW, M. H. L. (1986) *Nature*, **324**, 618–619.
12. ANDREWS, P. W. *et al* (1983) *in* Teratocarcinoma stem cells, eds. Silver, L. M., Martin, G. R. and Strickland, S., Cold Spring Harbor, New York, pp 579–590.
13. BRONSON, D. L. *et al* (1983) *ibid*, pp 597–605.
14. ANDREWS, P. W. *et al* (1984) *Lab.Invest.*, **50**, 147–162.
15. THOMPSON, S. *et al* (1984) *J.Cell.Sci.*, **72**, 37–64.
16. ANDREWS, P. W. (1984) *Develop.Biol.*, **103**, 285–293.
17. ASHCRAFT, K. W. and HOLDER, T. M. (1974) *J.Paediatric. Surg.*, **9**, 691–705.
18. LAZARIDES, E. (1980) *Nature*, **283**, 249–256.

## Figures and Tables

**Figure 1 f1:**
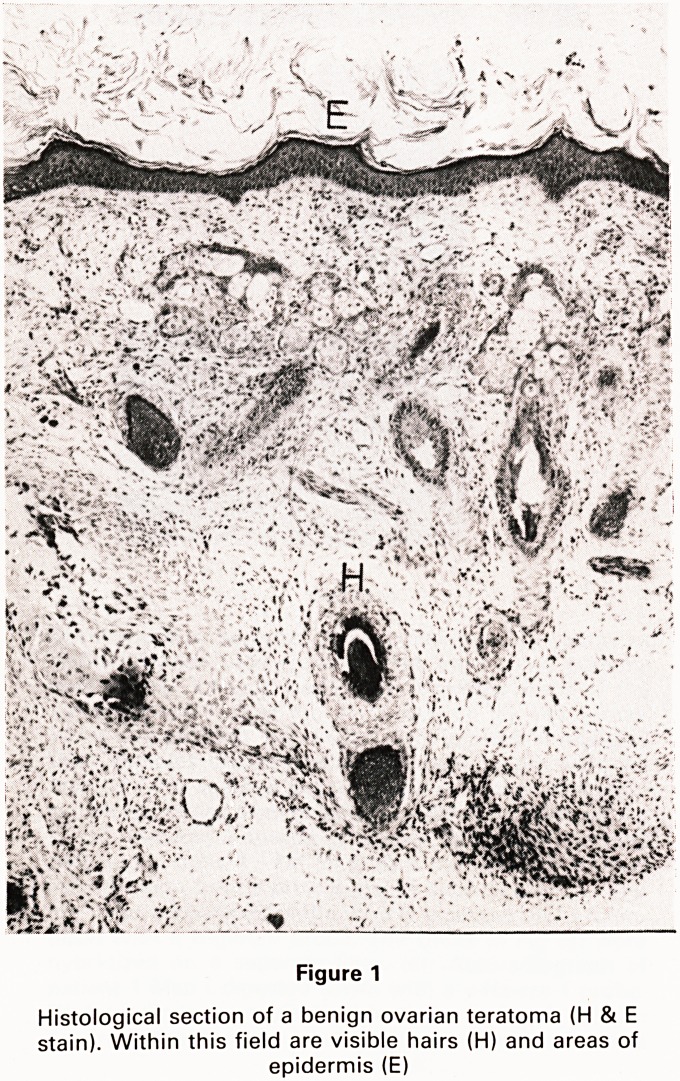


**Figure 2 f2:**
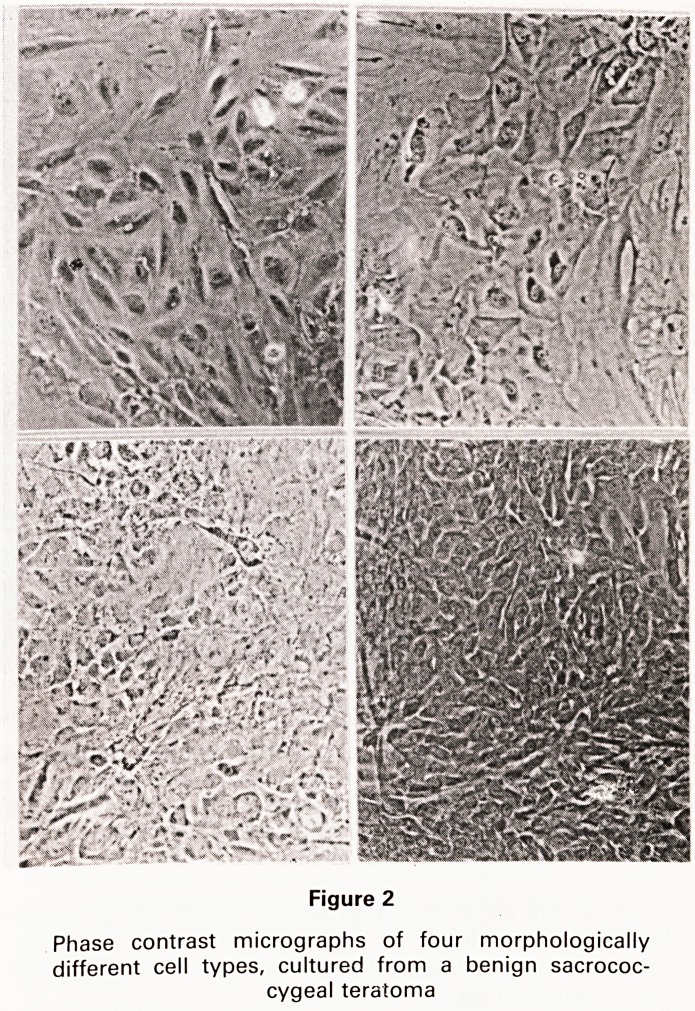


**Figure 3 f3:**